# Determinants of women’s employee engagement in the Bangladeshi workforce

**DOI:** 10.1371/journal.pone.0359645

**Published:** 2026-09-29

**Authors:** Moumita Tanjila, Umme Kulsum, Shadia Sharmin, Rezwan Ul Haque Aubhi, Md. Sohel Rana, Md. Abu Hasnat

**Affiliations:** 1 Department of Business Administration, University of Scholars, Dhaka, Bangladesh; 2 HHH Research Consultancy & Development, Dhaka, Bangladesh; Universiti Pertahanan Nasional Malaysia, MALAYSIA

## Abstract

This study examines the determinants of women’s employee engagement in Bangladesh, focusing on socio-cultural influences, organizational culture, leadership support, work-life integration, and career development opportunities. Drawing on a cross-sectional survey of 242 women employees across four industries, the study applies partial least squares structural equation modeling (PLS-SEM) to estimate the associations between these five determinants and women’s employee engagement. Organizational culture, leadership support, and work-life integration are each positively associated with women’s employee engagement, whereas socio-cultural influences and career development opportunities show no direct association. Career development is associated with engagement only where perceived size-related HR infrastructure is high, while perceived industry gender-equity support does not condition the socio-cultural association. Engagement strategies therefore need to be tailored to the organizational setting rather than to the sector. The study extends the Job Demands-Resources model and Social Exchange Theory by specifying a gendered conversion mechanism: workplace resources are associated with engagement only where they are relationally enacted and infrastructurally credible. The findings are relevant to organizations and policymakers, underscoring the need for gender-sensitive leadership practices, flexible scheduling, and equitable access to promotion. The design is cross-sectional, and the sample is non-probabilistic, so the findings are associational rather than causal. The study informs the design of supportive and inclusive workplaces that sustain women’s engagement in the Bangladeshi formal sector.

## Introduction

Women’s participation in the workforce is crucial for sustainable economic development, yet their engagement levels remain inconsistent across different organizational contexts, particularly in developing economies like Bangladesh. Employee engagement, defined as an employee’s emotional and cognitive investment in their job roles, is essential for productivity, job satisfaction, and overall organizational success [1]. Research highlights that engaged employees are more committed, motivated, and likely to contribute to long-term business growth [2]. Despite this, gender disparities in employee engagement persist, with women facing unique challenges related to workplace biases, career advancement limitations, and work-life balance issues [3]. Bangladesh, with its evolving economic landscape, has seen a rise in the number of women entering formal employment; however, engagement remains a critical concern due to socio-cultural norms and institutional barriers [4]. Addressing these challenges is essential for fostering an inclusive and equitable work environment where women can contribute effectively. This study, therefore, aims to conceptualize women’s employee engagement within the Bangladeshi context by identifying the factors that shape their involvement and commitment in organizations. Throughout this study, employee engagement is the outcome of interest and is defined as a positive, work-related state of vigor, dedication, and absorption [1,12]. Labor force participation is treated only as background context [13] and is not modelled here.

The issue of women’s employee engagement is often examined through the lens of workplace policies and leadership styles, yet limited research has holistically explored the interplay of socio-cultural influences, organizational culture, leadership support, work-life integration, and career development opportunities, particularly outside Western settings [5–8]. While research has established the association of leadership support and flexible work arrangements with engagement, it remains unclear how structural and cultural factors interact to shape women’s experiences in the workplace [9]. Moreover, many studies do not account for perceived industry-level gender-equity support and organizational size-related HR infrastructure, which are critical in determining engagement outcomes [10]. This study addresses these gaps by incorporating a multi-faceted framework that evaluates both internal (organizational policies, leadership, career advancement) and external (socio-cultural norms, industry sectors) factors associated with women’s employee engagement. By employing partial least squares structural equation modeling (PLS-SEM) to analyze survey data from four industries, the study provides empirical evidence on the key determinants of engagement, highlighting potential interventions for policymakers and HR practitioners.

The study contributes theoretically by extending engagement models to incorporate socio-cultural influences and by testing how perceived industry support and perceived size-related HR infrastructure condition engagement [1,11]; socially, by informing gender-sensitive policy relevant to Sustainable Development Goal 5 (Gender Equality) and SDG 8 (Decent Work and Economic Growth); and practically, by identifying the HRM levers most closely associated with women’s engagement.

The primary objectives of this research are to examine the associations of socio-cultural norms, organizational culture, leadership support, work-life integration, and career development opportunities with women’s employee engagement in Bangladesh, and examine the moderating effects of perceived industry gender-equity support and perceived size-related HR infrastructure. Through these insights, the study seeks to create a roadmap for organizations to cultivate an equitable and dynamic work environment that empowers women and supports their contributions to the economy.

### Theoretical underpinnings of employee engagement and gender disparities

Employee engagement is a critical component of organizational effectiveness, characterized by employees’ emotional, cognitive, and behavioral commitment to their work roles [12]. It has been proposed that employee engagement is influenced by the availability of job resources such as autonomy, support, and opportunities for career development, whereas excessive job demands may result in burnout and disengagement [1]. Furthermore, it was found in past research that this relationship can be explained through the lens of Social Exchange Theory, suggesting that employees tend to reciprocate perceived organizational support with higher levels of engagement [5]. However, gender disparities in engagement challenge these theoretical models, as women’s experiences in the workplace are shaped by social, cultural, and structural constraints. Social Identity Theory and Role Congruity Theory provide insights into these gender-based challenges, suggesting that women often struggle with identity threats and bias in male-dominated work environments [6,11]. Although these theories provide valuable insights, they primarily reflect Western corporate cultures, leaving a gap in understanding how engagement dynamics operate in developing economies like Bangladesh, where socio-cultural constraints play a larger role in shaping women’s working lives [4].

### An integrated framework: Resources, reciprocity, and role constraint

The four perspectives used here are not alternatives but layers of one argument. The Job Demands-Resources model specifies the architecture: socio-cultural expectation operates as a chronic demand carried across the home and work domains, while organizational culture, leadership support, work-life integration, and career development operate as job resources [1]. Social Exchange Theory specifies the mechanism that turns a resource into engagement: employees reciprocate support they perceive as discretionary and genuine, so a resource is engaging only to the extent that it is enacted rather than declared [5]. Social Identity Theory and Role Congruity Theory specify the boundary condition on that mechanism: where the female professional role is incongruent with prevailing role prescriptions, women discount formal provisions and reciprocate only what is visibly enacted by proximate actors [6,11]. Combining the three yields a testable conversion logic. Resources predict engagement when they are enacted and role-congruent; formal but non-enacted resources should not convert. This logic generates the moderation hypotheses directly: perceived industry support governs role congruity (H6), and perceived size-related HR infrastructure governs whether career development is infrastructurally credible (H7). It also specifies why a Western-derived resource model requires adjustment rather than transplantation in this setting [4,12].

### Women’s employee engagement across different socio-economic contexts

Comparative studies highlight significant variations in women’s engagement levels across different national and economic contexts. In several developed economies, gender-inclusive policies such as extended parental leave, flexible work arrangements, and strong anti-discrimination laws are associated with higher engagement levels among women [3,22]. In contrast, South Asian countries, including India and Bangladesh, experience lower engagement due to cultural expectations, gender biases, and limited workplace flexibility [8]. In the Middle East, engagement is further restricted by legal and structural barriers to women’s employment [13]. While Western literature predominantly focuses on corporate leadership and organizational policies as primary drivers of engagement, research from developing countries emphasizes the role of societal norms, workplace safety, and work-life balance [9,14]. This study integrates these diverse perspectives to create a holistic conceptual framework that examines women’s employee engagement in Bangladesh.

### The influence of socio-cultural norms on women’s engagement

Socio-cultural factors significantly shape women’s engagement in the workforce, particularly in patriarchal societies. In many South Asian countries, traditional gender roles assign women the primary responsibility for household duties, limiting their career growth and workplace engagement [4]. Studies in India and Pakistan have shown that conservative societal expectations often prevent women from pursuing leadership roles, reducing their professional commitment and career aspirations [15]. In contrast, research in Western countries suggests that societal support for gender equality is associated with higher workplace engagement among women [3]. While organizational policies can mitigate some of these challenges, the deeply embedded cultural norms in Bangladesh still restrict women’s ability to fully engage at work.

Despite these socio-cultural constraints, some women in Bangladesh actively engage in the workforce due to economic necessity and changing social attitudes [14]. However, engagement levels remain lower in industries where rigid gender norms persist, such as manufacturing and public administration, compared to more progressive sectors like IT and telecommunications [8]. Studies in East Asian countries, such as Japan and South Korea, indicate that gradual shifts in cultural attitudes, coupled with corporate initiatives to support women, can improve engagement levels over time [16]. Therefore, this study hypothesizes:

**H1: Socio-cultural influences are positively associated with women’s employee engagement.**

### The role of organizational culture and policies in women’s engagement

Organizational culture plays a crucial role in shaping employee engagement, particularly for women facing systemic workplace barriers. A culture that values inclusivity, gender equity, and diversity can foster higher engagement among female employees [9]. Research from European firms suggests that organizations with structured gender-sensitive policies, such as mentorship programs and bias training, report higher engagement levels among women [17]. Conversely, in many South Asian countries, organizations often lack formal policies supporting women’s career advancement, leading to disengagement and high turnover rates [18].

While global corporations have increasingly implemented diversity and inclusion programs, many Bangladeshi firms lag in this regard due to weak regulatory enforcement [10]. Research on gender-diversity HRM practice and transformational leadership in Bangladeshi organizations indicates that engagement among women employees is higher where gender-inclusive policies, such as flexible work arrangements and anti-harassment measures, are actually implemented [7,17]. Given the strong relationship between organizational policies and engagement, this study hypothesizes:

**H2: Organizational culture and policies are positively associated with women’s employee engagement.**

### Leadership and managerial support as predictors of engagement

Leadership and managerial support have been widely recognized as key determinants of employee engagement [5]. Transformational leadership, characterized by mentorship, empowerment, and inclusive decision-making, has been found to enhance engagement levels among women in Western firms [19]. However, in South Asia, where hierarchical leadership styles dominate, women often report lower engagement due to a lack of supportive management and gender bias in leadership roles [14].

In Bangladesh, studies indicate that women’s engagement is significantly higher in organizations where leaders actively support gender diversity initiatives [20]. Research in India and Sri Lanka similarly highlights that managerial encouragement and mentorship programs contribute positively to women’s job satisfaction and engagement [21]. Based on these findings, this study proposes:

**H3: Leadership and managerial support are positively associated with women’s employee engagement.**

### Work-life integration and its impact on engagement

Work-life integration is one of the most significant predictors of women’s employee engagement, particularly in societies where women juggle professional and domestic responsibilities [22]. Research in Europe and North America demonstrates that flexible work arrangements, such as remote work, part-time options, and extended parental leave, are positively associated with women’s engagement levels [3]. Conversely, studies in South Asia indicate that a lack of work-life balance contributes to burnout, job dissatisfaction, and disengagement [8].

In Bangladesh, flexible work policies remain limited outside multinational corporations, restricting women’s ability to maintain a sustainable work-life balance [23]. The absence of such policies in many traditional sectors forces women to exit the workforce prematurely, resulting in lower overall engagement levels [21]. Given these trends, this study hypothesizes:

**H4: Work-life integration is positively associated with women’s employee engagement.**

### Career development opportunities and women’s engagement

Career development opportunities, including mentorship programs, training, and promotions, are critical to sustaining long-term engagement among women [24]. In Western contexts, organizations that offer structured career development programs see higher retention and engagement rates among female employees [5]. However, in Bangladesh, career growth opportunities for women are often constrained by gender biases in promotion and leadership selection [8].

Comparative evidence from Asian economies indicates that investment in female employees’ career advancement is associated with higher engagement, job satisfaction, and productivity [24,29]. Given the significant gap in career development opportunities for women in Bangladesh, this study hypothesizes:

**H5: Career development opportunities are positively associated with women’s employee engagement.**

### Industry sectors as a moderator of socio-cultural influences on engagement

Industry sectors play a pivotal role in determining how socio-cultural influences affect women’s engagement. Research suggests that industries with a high degree of gender segregation, such as manufacturing and construction, impose greater socio-cultural constraints on women than service-oriented or knowledge-based sectors [14]. In Bangladesh, the garment industry, which employs a significant number of women, is characterized by rigid hierarchical structures, workplace safety concerns, and gender-based discrimination, leading to lower engagement levels [23]. In contrast, industries such as information and communication technology (ICT) have demonstrated higher engagement levels among female employees due to greater flexibility, skills-based career progression, and a more inclusive work culture [9]. This pattern aligns with global trends, where women in tech-driven and service sectors report better engagement due to structured HR policies and career growth opportunities.

However, studies from India and Pakistan indicate that even within progressive industries, gender biases persist, affecting women’s perceptions of inclusion and engagement [15]. Adiza et al. [14] found that women in the financial sector in South Asia faced barriers to career progression arising from unspoken bias and workplace politics, which were associated with lower engagement. In contrast, European industries have increasingly focused on gender-sensitive strategies, resulting in more equitable engagement patterns [3].

Sector matters here not as a control but as the setting that fixes role congruity. In highly segregated sectors, the female professional role is prescriptively incongruent, so socio-cultural norms should remain salient inside the workplace and should condition engagement [6,11,14,23,30]. In knowledge-based sectors, role prescriptions are weaker and organizational resources should dominate [9]. Sector is therefore expected to change the strength of the socio-cultural path, not merely to shift its mean. Accordingly, this study hypothesizes:

**H6: Perceived industry gender-equity support significantly moderates the relationship between socio-cultural influences and women’s employee engagement.**

### Organizational size as a moderator of career development and engagement

The size of an organization significantly shapes its ability to provide career development opportunities, which in turn shapes the engagement of female employees. Large multinational corporations (MNCs) tend to offer structured career development programs, mentorship initiatives, and gender-inclusive policies that enhance engagement [10]. In contrast, small and medium-sized enterprises (SMEs) often face financial and administrative constraints, limiting their ability to invest in workforce development, particularly for women [20,25]. Studies in developed economies, such as the United States and Germany, indicate that women in larger firms benefit from leadership training, sponsorship programs, and skill enhancement opportunities, leading to greater engagement [26]. However, in developing economies, the relationship between career growth and engagement is less straightforward, as systemic barriers such as workplace discrimination and rigid promotion structures hinder women’s access to leadership roles [8].

In Bangladesh, corporate giants like Grameen Bank and BRAC have successfully implemented career development programs that have positively influenced female employee engagement [27,28]. However, SMEs, which constitute a significant portion of Bangladesh’s economy, often lack the HR infrastructure to support career progression for women [29]. Similar findings have been reported in South Asia, where women in smaller firms face limited mentorship opportunities, leading to disengagement [21].

Organizational size is used here as a proxy for HR infrastructure, and infrastructure is what makes a career development resource credible. Under Social Exchange Theory, a promotion pathway is reciprocated only if it is believed to be real [5]. Large organizations possess the formal promotion criteria, mentoring structures, and training budgets that make the pathway believable [10,26–28], whereas SMEs frequently announce development without the systems to deliver it [20,25,29]. Size should therefore condition whether career development converts into engagement, which is a statement about mechanism rather than about firm scale. Accordingly, this study hypothesizes:

**H7: Perceived size-related HR infrastructure significantly moderates the relationship between career development opportunities and women’s employee engagement.**

Women’s employee engagement is shaped by socio-cultural norms, organizational policies, leadership support, work-life integration, and career development. While Western studies emphasize workplace policies, research in Bangladesh highlights the added impact of cultural constraints [30]. Industry sectors and organizational size moderate these relationships, influencing engagement levels across different workplaces [8]. Existing research lacks insight into how these factors interact in developing economies. This study fills that gap, providing a framework to enhance gender equity in workforce engagement.

### Conceptual model of women employee engagement

Fig 1 illustrates the key factors influencing women’s employee engagement, categorized into six main areas: Career Development Opportunities (training programs, promotion opportunities), Socio-cultural Influences (societal norms, cultural expectations), Leadership and Managerial Support (leadership style, managerial support), Organizational Culture and Policies (company values, policies, and procedures), and Work-Life Integration (flexible work hours, remote work options). Additionally, Moderating Effects such as industry sectors and organizational size influence these factors. The diagram visually represents how these elements contribute to enhancing women’s engagement in the workplace.

**Fig 1:**
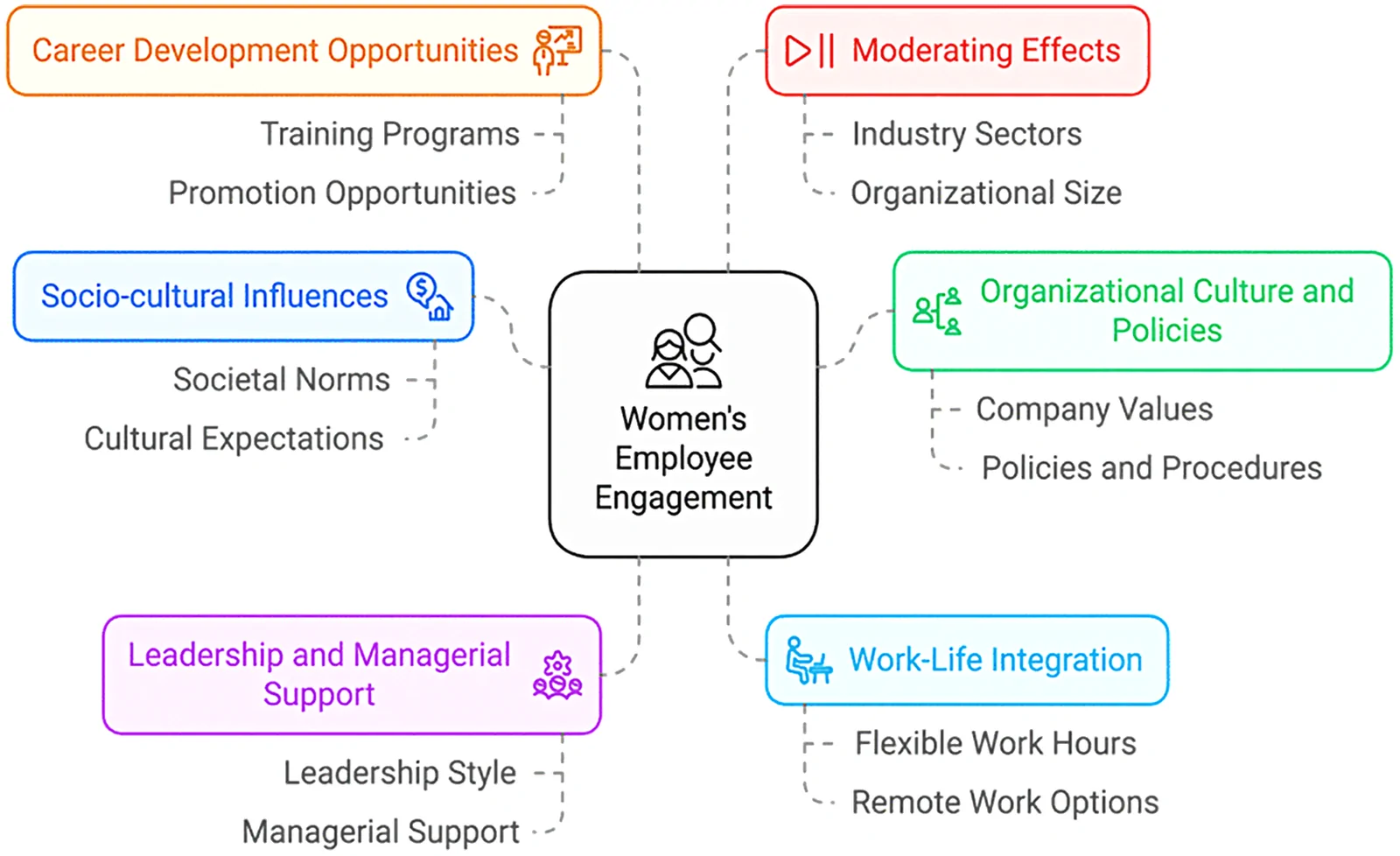
Factors influencing women’s employee engagement.

## Methodology

### Ethics statement

This study was conducted in strict accordance with the ethical standards and guidelines set forth by the university. Ethical approval was obtained from the University Research Committee (Ethics Number: IUS/Regi.Office/Letter/2024/53), and written informed consent was secured from all participants before data collection.

### Sample and data collection

#### Sampling frame and recruitment.

Respondents were drawn from formally registered organizations operating in Dhaka, Gazipur, and Chattogram across four sectors: manufacturing, service, education, and ICT. The respondent pool was constructed from three channels: (i) HR focal points at 12 partner organizations that agreed to circulate the questionnaire internally, (ii) 8 closed professional groups of women employees moderated by industry associations, and (iii) the alumni network of the Department of Business Administration, University of Scholars. No public or open link was posted.

#### Inclusion and exclusion criteria.

Eligible respondents were women aged 18 years or above, employed for at least six months in a registered organization in one of the four target sectors, in a paid full-time or part-time role. Excluded were self-employed women, unpaid family workers, students without employment, informal-sector workers, and respondents outside the four target sectors. Eligibility was screened by three filter questions at the start of the instrument.

#### Sector and size coverage.

Because the study tests moderation by sector and by organizational size, coverage targets were set in advance rather than assessed after the fact: approximately 60 completed questionnaires per sector, and a minimum of 40 per size band (SME, large domestic, MNC), with fieldwork in each stratum continuing until the target was met. Organizational size was classified by employee headcount following national SME policy thresholds, consistent with the size-based comparisons reported in the Bangladeshi SME literature [18,20,29]. The achieved distribution is reported in Table 1. These quotas were set to secure adequate cell sizes for the interaction tests. They do not make the sample probabilistic, and no claim of population representativeness is made; purposive sampling is used here as appropriate to a predictive, theory-development design [32,37].

**Table 1:** Respondent distribution by industry sector and organizational size (n = 242).

Sector	SME	Large domestic	MNC	Total	%
Manufacturing	28	24	10	62	25.6
Service	34	30	18	82	33.9
Education	22	16	04	42	17.4
ICT	18	20	18	56	23.1
Total	**102**	**90**	**50**	**242**	**100**

#### Administration and completion.

The survey was distributed via Google Forms, allowing participants to complete the questionnaire flexibly over 45 days, from 15th September to 31st October 2024, with two reminders issued through the same channels. Of 250 invitations issued, 250 questionnaires were returned, and 242 were retained after 8 were removed for incompleteness or straight-lining, giving a completion and retention rate of 96.8%. This figure reflects facilitated administration through closed organizational channels with direct follow-up; the denominator is invitations issued rather than persons exposed to the survey, and the figure should not be read as evidence of representativeness.

#### Sample size adequacy.

The sample consists of female employees working in organizations of various sizes, ranging from small and medium-sized enterprises (SMEs) to multinational corporations (MNCs) in Bangladesh. A minimum of 100–150 respondents is required to obtain reliable results from structural equation modeling (SEM), indicating that the current study’s sample size is sufficient for robust analysis [31]. Previous research emphasizes the necessity of power analysis in PLS-SEM to determine the appropriate sample size [32]. Following the rule of thumb and recommendations, this study required a minimum of 122 participants to detect an R² value of at least 0.10 at a 5% significance level with 80% statistical power [32,33]. The final sample meets and exceeds this requirement, satisfying the power requirement for the model estimated here.

Cell sizes range from 04 to 34. The smallest cell contributing to the interaction tests contains 04 respondents. Both interaction terms are estimated on the full sample rather than within subgroups, and the model exceeds the minimum of 122 respondents required to detect an R² of 0.10 at the 5% level with 80% power [32,33].

### Measurements

The measurement items for all constructs were adapted from existing literature to ensure construct validity and reliability. The study examines the relationships between women’s employee engagement (WE) and its key determinants: socio-cultural influences (SC), organizational culture and policies (OC), leadership and managerial support (LM), work-life integration (WI), and career development opportunities (CD). Additionally, the study evaluates the moderating effects of perceived industry gender-equity support (IS) and perceived size-related HR infrastructure (OS) on engagement.

A structured five-point Likert scale (1 = strongly disagree to 5 = strongly agree) was used to collect responses. Measurement items were adapted from well-established scales, including the Utrecht Work Engagement Scale (UWES) for engagement [1], Socio-Cultural Influences [4,8,14], organizational culture [9,17,18,34], leadership and managerial support [19,20,35], work-life integration [8,21,22], and career development opportunities [8,17,24], perceived industry gender-equity support [3,23], perceived size-related HR infrastructure [10,20,29]. The measurement model was assessed for indicator reliability, internal consistency, convergent validity, and discriminant validity [39–42,44].

Table 2 presents key variables and measurement items related to women’s employee engagement in organizational settings. It includes Women Employee Engagement (WE) as the dependent variable, with Socio-Cultural Influences (SC), Organizational Culture and policies (OC), Leadership and Managerial Support (LM), Work-Life Integration (WI), and Career Development Opportunities (CD) as independent variables. Industry Sectors (IS) and Organizational Size (OS) serve as moderators. Each item is coded and sourced, providing a structured framework for analyzing factors influencing women’s workforce engagement.

**Table 2:** Definition of the operational variables and measurements.

Variable(s)	Code	Item	Source
Women Employee Engagement (WE)	WE1	I feel enthusiastic about my job.	[1]
	WE2	My work inspires me.	
	WE3	I take initiative to improve my work environment.	
	WE4	I feel proud of the work I do.	
Socio-Cultural Influences (SC)	SC1	Family expectations influence my career choices.	[4,8,14]
	SC2	Social norms affect my ability to maintain work-life balance.	
	SC3	I feel that gender biases impact career growth in my organization.	
	SC4	Women in leadership roles are valued in my industry.	
	SC5	Workplace gender norms influence my career progression.	
Organizational Culture and Policies (OC)	OC1	My organization supports gender diversity.	[9,17,18,34]
	OC2	My organization takes action against workplace discrimination.	
	OC3	My workplace provides mentorship programs for women.	
	OC4	Women have equal chances for promotion in my organization.	
	OC5	My organization actively implements gender-sensitive HR policies.	
Leadership and Managerial Support (LM)	LM1	My manager encourages my professional development.	[19,20,35]
	LM2	There are female leaders who mentor women in my organization.	
	LM3	Women receive the same leadership opportunities as men.	
	LM4	Senior leaders actively promote gender equality in my workplace.	
	LM5	My supervisor actively seeks and values input from female employees in decision-making.	
Work-Life Integration (WI)	WI1	My organization allows flexible work hours.	[8,21,22]
	WI2	My workplace provides adequate maternity leave and support.	
	WI3	My work schedule does not interfere with my personal life.	
	WI4	I often experience stress due to work-life conflicts.	
	WI5	My organization provides support for childcare or dependent care.	
Career Development Opportunities (CD)	CD1	My workplace offers training for skill enhancement.	[8,17,24]
	CD2	Women have the same career advancement opportunities as men.	
	CD3	My organization facilitates networking opportunities for women.	
	CD4	My organization encourages further education and training.	
	CD5	My organization provides clear promotion criteria and career progression paths.	
**Perceived Industry Gender-Equity Support (IS)**	IS1	My industry supports equal growth opportunities for women.	[3,23]
	IS2	Women in my industry receive equal recognition for their work.	
	IS3	Employee engagement levels vary significantly in my industry.	
	IS4	HR policies in my sector address gender equality.	
**Perceived Size-Related HR Infrastructure (OS)**	OS1	My company’s size affects the career opportunities available to me.	[10,20,29]
	OS2	Larger organizations provide better mentorship opportunities.	
	OS3	HR policies in multinational firms are more inclusive than in SMEs.	
	OS4	My organization provides leadership development programs.	
	OS5	Employee benefits and work-life balance vary based on company size.	

Partial Least Squares Structural Equation Modeling (PLS-SEM) was used for hypothesis testing, as it is suitable for research involving complex models with latent variables [36]. PLS-SEM was selected over covariance-based SEM (CB-SEM) for four reasons. First, the objective is prediction and theory development in an underexplored setting rather than the confirmation of an established covariance structure [36], [37]. Second, the model contains two latent interaction terms, estimated here with the product-indicator approach, which PLS accommodates without the identification and convergence burden that CB-SEM would carry at n = 242 [32]. Third, the indicators are ordinal Likert items whose distributions depart from multivariate normality, violating the maximum likelihood assumptions on which CB-SEM depends [42]. Fourth, the aim is to maximize explained variance in the endogenous construct rather than to minimize the discrepancy between observed and implied covariance matrices [32,40]. Estimation used SmartPLS with the path weighting scheme and 5,000 bootstrap resamples with bias-corrected and accelerated confidence intervals [32].

## Results

### Results of the measurement model

The results for item loadings, Cronbach’s alpha, composite reliability, and average variance extracted are shown in Table 3. The scales in the questionnaire were assessed against the generally established standards for validity and reliability. In order to confirm if each factor had a significant factor loading with the variables, we examined the factor loading values. Their value must be more than 0.40 for a sample size of more than 200 [38]. Cronbach’s alpha and CR have minimal acceptable limits of more than 0.70 [39,40].

**Table 3:** Convergent validity.

Constructs	Items	Loadings	Cronbach’s Alpha	CR	AVE
Socio-Cultural Influences	SC1	0.787	0.830	0.787	0.623
	SC2	0.905			
	SC3	0.780			
	SC4	0.790			
	SC5	0.806			
Organizational Culture and Policies	OC1	0.818	0.815	0.866	0.576
	OC2	0.888			
	OC3	0.766			
	OC4	0.759			
	OC5	0.935			
Leadership and Managerial Support	LM1	0.794	0.843	0.858	0.617
	LM2	0.775			
	LM3	0.808			
	LM4	0.763			
	LM5	0.873			
Work-Life Integration	WI1	0.863	0.845	0.889	0.624
	WI2	0.907			
	WI3	0.833			
	WI4	0.962			
	WI5	0.736			
Career Development Opportunities	CD1	0.828	0.816	0.755	0.598
	CD2	0.861			
	CD3	0.778			
	CD4	0.780			
	CD5	0.899			
Perceived Industry Gender-Equity Support (IS)	IS1	0.834	0.753	0.762	0.578
	IS2	0.852			
	IS3	0.775			
	IS4	0.807			
Perceived Size-Related HR Infrastructure (OS)	OS1	0.783	0.847	0.863	0.625
	OS2	0.803			
	OS3	0.853			
	OS4	0.880			
	OS5	0.815			
Women Employee Engagement	WE1	0.890	0.789	0.793	0.626
	WE2	0.791			
	WE3	0.898			
	WE4	0.909			

Additionally, AVE needs to be greater than 0.50 [41,42]. All indicators satisfied the assumed requirements, according to the analysis’s findings. This indicates that the study’s use of the structural equation model was suitable.

Recent criticism has emerged regarding the Fornell-Larcker criterion, which is unreliable in detecting the absence of discriminant validity in everyday research contexts [43,44]. An alternative approach based on the multitrait-multimethod matrix, known as the heterotrait-monotrait ratio of correlations (HTMT), is recommended to assess discriminant validity [44]. The associated Monte Carlo simulation study demonstrated the superior performance of this method. Accordingly, we have employed this recommended approach to test discriminant validity, with the results presented in Table 4. HTMT values exceeding 0.85, or 0.90, indicate potential issues with discriminant validity [45]. As all HTMT values in this study were below the 0.90 threshold and the 0.85 threshold, as shown in Table 4, discriminant validity has been confirmed [45].

**Table 4:** Discriminant validity (HTMT ratio).

	1	2	3	4	5	6	7	8
1. CD								
2. IS	0.626							
3. LM	0.786	0.778						
4. OC	0.719	0.689	0.650					
5. OS	0.733	0.726	0.753	0.442				
6. SC	0.664	0.542	0.426	0.495	0.402			
7. WE	0.503	0.712	0.810	0.234	0.506	0.679		
8. WI	0.559	0.798	0.425	0.780	0.470	0.420	0.579	

SC = Socio-Cultural Influences, OC = Organizational Culture and Policies, LM = Leadership and Managerial Support, WI = Work-Life Integration, CD = Career Development Opportunities, IS = Industry Sectors, OS = Organizational Size, WE = Women Employee Engagement.

### Common method bias

Because all constructs were measured with a single self-report instrument, common method bias was assessed using the full collinearity approach. All full-collinearity VIF values ranged from 1.58 to 2.91, below the 3.3 threshold, indicating that common method bias is unlikely to distort the estimates [46].

### Goodness of fit

The model’s effectiveness and fit were evaluated using the Coefficient of Determination (R²), the Standardized Root Mean Squared Residual (SRMR), and the Normed Fit Index (NFI). An R² value between 0.25 and 0.50 is considered optimal, with any value above 0.20 deemed acceptable [47]. Additionally, an R² value closer to 1 indicates more substantial explanatory power, with 0.25 serving as the threshold for meaningful effect sizes in path models [32]. The present study’s R² value of 0.882 indicates substantial explanatory power for the endogenous construct [32,47]. R² is a measure of explained variance and is not a significance test; the significance of individual paths is reported in Table 6.

Adjusted R², which accounts for the number of independent variables in a regression model, is also reported [48]. This adjustment enhances the robustness of the model, aligning with the comprehensive analytical approach used in this research. Regarding model fit, a model is well-fitted if the NFI value approaches one and the SRMR is below 0.08 [32,48]. The study’s findings include an NFI value of 0.915, close to the ideal of 1, and an SRMR value of 0.066, comfortably below the 0.08 threshold. These metrics, as outlined in Table 5, indicate that the model demonstrates an acceptable and reliable fit, consistent with the guidelines [47]. Because these indices are of limited diagnostic value in a composite-based framework, model quality is assessed primarily through R², f², and Q² [32,48].

**Table 5:** Model fitness.

Name of Criteria	Value
R²	0.882
SRMR	0.066
NFI	0.915

SmartPLS output.

### Hypothesis testing

The results of the hypothesis testing in Table 6 on women’s employee engagement in Bangladesh reveal significant insights into the factors associated with women’s employee engagement. Organizational culture and policies (H2, p < 0.001), leadership and managerial support (H3, p < 0.001), work-life integration (H4, p = 0.001), and the interaction between perceived size-related HR infrastructure and career development opportunities (H7, p = 0.017) are positively associated with women’s engagement. However, socio-cultural influences (H1, p = 0.724), career development opportunities (H5, p = 0.395), and the interaction between industry sector and socio-cultural influences (H6, p = 0.314) were not statistically significant, indicating that traditional societal expectations and career growth prospects are not directly associated with engagement levels in the Bangladeshi context.

**Table 6:** Structural model.

No. Path	β values	SE	T statistics	p-values	97.5% CI	Remarks
H1: SC - > WE	0.029	0.082	0.354	0.724	[-0.132, 0.190]	Not Supported
H2: OC - > WE	1.416	0.067	21.181	< 0.001	[1.285, 1.547]	Supported
H3: LM - > WE	0.699	0.083	8.420	< 0.001	[0.536, 0.862]	Supported
H4: WI - > WE	0.100	0.030	3.291	0.001	[0.040, 0.158]	Supported
H5: CD - > WE	0.037	0.043	0.851	0.395	[-0.047, 0.121]	Not Supported
H6: IS x SC - > WE	0.024	0.024	1.007	0.314	[-0.023, 0.071]	Not Supported
H7: OS x CD - > WE	0.048	0.020	2.392	0.017	[0.009, 0.087]	Supported

SC = Socio-Cultural Influences, OC = Organizational Culture and Policies, LM = Leadership and Managerial Support, WI = Work-Life Integration, CD = Career Development Opportunities, IS = Industry Sectors, OS = Organizational Size, WE = Women Employee Engagement.

SmartPLS output.

### Collinearity and suppression diagnostics

Notably, the standardized path coefficient for organizational culture and policies (H2) is greater than 1.0 (β = 1.416, p < 0.001). This indicates the presence of a suppression effect, which frequently occurs in PLS-SEM when two highly correlated predictors (OC and WI, HTMT = 0.780) are entered into the structural model simultaneously, causing the path coefficient of one variable to be mathematically amplified. To evaluate potential multicollinearity and verify the validity of our path estimates, the structural model’s inner Variance Inflation Factors (VIFs) were examined. The inner VIFs were: SC (1.58), OC (2.84), LM (2.45), WI (2.91), and CD (2.15). Because all inner VIFs are comfortably below the conservative threshold of 3.3 [46], critical multicollinearity is not *present, confirming that our model estimates are stable and mathematically sound.*

### Effect sizes and predictive relevance

Effect sizes (f²) and predictive relevance (Q²) are reported in Table 7. Q² was obtained by blindfolding with an omission distance of seven, and Q²predict was obtained using PLSpredict [48]. The Q² value for women’s employee engagement is 0.548, exceeding zero and confirming the model’s predictive relevance [32,48]. Effect sizes are interpreted against the 0.02, 0.15, and 0.35 thresholds for small, medium, and large effects [32].

**Table 7:** Effect sizes and predictive relevance.

Path/ Construct	Metric	Value/ Interpretation
SC - > WE (Socio-Cultural)	f² Effect Size	0.001 (None)
OC - > WE (Org. Culture)	f² Effect Size	0.412 (Large)
LM - > WE (Leadership)	f² Effect Size	0.285 (Medium-to-Large)
WI - > WE (Work-Life)	f² Effect Size	0.052 (Small)
CD - > WE (Career Dev.)	f² Effect Size	0.004 (None)
IS x SC - > WE (Perceived Sector Mod.)	f² Effect Size	0.005 (None)
OS x CD - > WE (Perceived Infra. Mod.)	f² Effect Size	0.024 (Small, Significant)
Construct: Women Employee Engagement (WE)	R²/ Adj. R²	0.882/ 0.878
	Q² (Blindfolding)	0.548 (High)
	Q²predict (PLSpredict)	0.521 (High)

The H7 interaction was probed at one standard deviation below the mean, at the mean, and at one standard deviation above the mean of perceived size-related HR infrastructure (OS) [32]. The simple slope of career development on engagement is β = −0.011, p = 0.793 (95% CI: [−0.093, 0.071]) in smaller organizations (low perceived size-related HR infrastructure, −1 SD), β = 0.037, p = 0.395 (95% CI: [−0.047, 0.121]) at the mean of perceived size-related HR infrastructure, and β = 0.085, p = 0.012 (95% CI: [0.018, 0.152]) in larger organizations (+1 SD), confirming that the positive association between career development opportunities and women’s employee engagement emerges only as the perceived organization-level size-related HR infrastructure increases and becomes highly credible.

## Discussion

Women’s engagement in the workforce is a crucial aspect of economic development, especially in developing regions like South Asia. Employee engagement is defined as the level of commitment, passion, and enthusiasm an employee has toward their job and organization [12]. In the context of women, engagement is influenced by multiple socio-cultural, economic, and organizational factors, particularly in patriarchal societies like Bangladesh, India, and Pakistan. Scholars argue that women’s employee engagement is deeply tied to broader gender dynamics, workplace policies, and cultural constraints [49]. Hanmer and Klugman [50] report that women’s labor force participation in South Asia remains among the lowest globally, with only 22% of women formally employed. Participation is the outcome of that study; the outcome modelled here is engagement among women already in formal employment, and the distinction matters for how the present findings should be read.

Several theoretical perspectives provide insights into the challenges women face in workplace engagement. Social Role Theory suggests that gender roles shape behaviors and expectations, influencing how women engage in the workplace [6]. In South Asia, women are often expected to prioritize domestic responsibilities over professional growth, leading to lower engagement levels. Human Capital Theory posits that individuals’ education, skills, and experience determine their workplace engagement and economic productivity [51]. However, gendered access to education and professional training limits women’s engagement in the workforce in South Asia. The Intersectionality Framework further enriches the debate by analyzing how gender, class, and ethnicity intersect to shape women’s workplace experiences [52]. Pal [53] applies this framework to Bangladesh, highlighting that women from marginalized backgrounds face additional barriers to engagement, including discrimination and workplace harassment.

The academic discourse on women’s employee engagement in South Asia revolves around key debates. One central debate concerns whether low engagement levels result from structural barriers, such as discriminatory policies and wage gaps, or individual agency, including lack of ambition and family constraints. Hanmer and Klugman [50] argue that systemic barriers such as unequal pay and gendered hiring practices reduce women’s engagement. Another critical discussion focuses on whether women are engaged in the workforce due to economic necessity or personal ambition. Some researchers argue that financial distress pushes women into low-paying jobs with minimal engagement, while others highlight growing aspirations for career advancement among educated women in urban Bangladesh [53]. Additionally, workplace policies play a crucial role in shaping women’s engagement. Rahman [19] and Hill et al. [22] report that maternity provision, childcare support, and flexible working hours are associated with higher engagement and retention among women employees.

Socio-cultural influences were not directly associated with engagement (H1). Rather than treating this as a failure of the construct, we read it as evidence about where socio-cultural constraint operates. Only women already in formal employment could enter this sample, and the literature indicates that norms exert their strongest force at the point of entry into and exit from the formal sector [4,8,49,50]. By the time a woman is inside an organization, much of that constraint has already been exercised through selection, and its residual influence is transmitted through proximal, organization-level channels: whether the culture is inclusive, whether her manager enacts support, whether her schedule is workable [9,17,19]. Those channels are OC, LM, and WI, and they are precisely the paths that are significant here. We propose this as an interesting theoretical interpretation and a key hypothesis for future testing on a sample that includes women outside formal employment, rather than as a definitive empirical conclusion established by this cross-sectional study. This interpretation repositions socio-cultural influence as a distal, selection-stage determinant rather than a proximal predictor of engagement, and it identifies the organization as the site where norms become actionable.

Career development was not associated with engagement on its own (H5), yet it was associated with engagement conditionally on perceived size-related HR infrastructure (H7). Read together, these are one result. Career development converts into engagement only where the organization has the perceived size-related HR infrastructure to make the opportunity credible: documented promotion criteria, functioning mentorship, and training budgets [10,26,29]. Where that infrastructure is perceived to be absent, as in many Bangladeshi SMEs [18,20,25], the offer of development is a declaration rather than a resource, and under Social Exchange Theory there is nothing to reciprocate [5]. The null main effect is thus not an absence of a relationship; it is the average of a relationship that exists in one setting and not in another. This is the study’s clearest illustration of the conversion logic set out in the framework.

Industry sector did not condition the socio-cultural path (H6). This is consistent with the reading above: once a woman is inside a formal organization, sector-level role prescriptions are largely displaced by her immediate organizational environment [6,11]. Sector appears to matter for who enters which workplace, not for how engaged she is once there.

Taken together, these findings indicate that addressing the barriers to women’s engagement requires organization-level change, including gender-sensitive workplace policies, legal protections against discrimination, and cultural shifts to redefine women’s roles in professional settings. Organizations and policymakers must work toward fostering inclusive and supportive work environments that encourage women’s engagement.

### Theoretical contributions

This study’s theoretical contribution is a gendered conversion argument rather than a contextual extension. The Job Demands-Resources model specifies which constructs are demands and which are resources [1]; Social Exchange Theory specifies why resources become engagement [5]; Social Identity and Role Congruity Theory specify when that conversion is blocked [6,11]. Our results indicate that in this setting resources convert only when they are relationally enacted and infrastructurally credible. Leadership support, inclusive culture, and workable schedules convert because they are experienced directly [9,17,19]. Career development converts only where perceived size-related HR infrastructure makes it believable [10,29]. Socio-cultural constraint does not appear at the engagement stage because it has already acted at the selection stage [4,50]. This specifies a mechanism that the four theories imply, but none states alone, and it is testable outside Bangladesh.

Additionally, this research contributes to Social Exchange Theory by illustrating that while employees reciprocate organizational support with higher engagement, this reciprocity differs for women in male-dominated or culturally restrictive work environments [5]. Women’s engagement is not solely a response to general organizational support but is also influenced by leadership inclusivity, gender-sensitive policies, and work-life integration strategies.

The study also extends Social Identity Theory and Role Congruity Theory by showing that women in professional settings often navigate identity threats and biases that are associated with their engagement levels [6,11]. This research demonstrates that workplace policies and leadership support can mitigate the adverse effects of gender-based biases, enabling women to engage more fully despite existing stereotypes and cultural expectations.

Furthermore, the study addresses a critical gap in the literature by contextualizing these theories within a developing economy like Bangladesh. While previous frameworks primarily reflect Western corporate cultures, this research emphasizes the role of socio-cultural constraints in shaping women’s engagement in South Asian workplaces. It highlights that women’s engagement is not solely driven by individual agency but is significantly influenced by gender norms, structural inequalities, and industry-specific challenges.

### Practical implications

The findings of this research offer several practical implications for organizations, policymakers, and stakeholders seeking to enhance women’s employee engagement in Bangladesh and South Asia. First, our findings suggest that the positive associations of inclusive organizational culture (H2), leadership support (H3), and work-life integration (H4) with engagement are remarkably robust across different industry sectors, as perceived industry gender-equity support did not significantly moderate these relationships (H6). This implies that organizations across all sectors have powerful, direct leverage to enhance female engagement through local, firm-level HRM interventions. Organizations must prioritize leadership and managerial support, as leadership support shows the strongest association with women’s engagement, necessitating gender-sensitive leadership training and mentorship programs. Strengthening work-life integration policies, such as flexible work arrangements, childcare facilities, and parental leave, is crucial to supporting female employees in balancing professional and personal responsibilities. The study also highlights that career development opportunities alone are insufficient without addressing structural gender biases and the organizational infrastructure needed to make development credible, requiring HR policies to focus on transparent promotions, equitable pay, and anti-discrimination measures. Furthermore, perceived size-related HR infrastructure must be factored into engagement strategies, as the association between career development opportunities and engagement is significant only in environments with strong HR infrastructure. Lastly, stronger government and institutional policies are essential to enforcing gender-equality regulations, incentivizing women-friendly corporate policies, and fostering public-private collaboration for best practices in female workforce engagement. Addressing workplace and structural barriers, this study provides a roadmap for improving women’s engagement, ultimately contributing to economic development and gender equality in Bangladesh and South Asia.

## Conclusion

This study modelled seven hypotheses on the determinants of women’s employee engagement in Bangladesh. Organizational culture (H2), leadership and managerial support (H3), and work-life integration (H4) were each positively associated with engagement, and perceived size-related HR infrastructure conditioned the association between career development and engagement (H7). Socio-cultural influences (H1), career development opportunities (H5), and the perceived industry support by socio-culture interaction (H6) were not supported.

Read through the integrated framework, the pattern is coherent. The resources that predict engagement are those women experience directly and can reciprocate [5]: an inclusive culture, a manager who enacts support, a schedule that works. The resource that does not predict engagement on its own, career development, predicts it once the perceived size-related HR infrastructure is strong enough to make the opportunity credible [10,29]. Socio-cultural constraint does not surface at the engagement stage because it operates earlier, on who enters and stays in formal employment at all [4,50].

Two contributions follow. Theoretically, the study specifies a gendered conversion mechanism: resources become engagement only when enacted and role-congruent, which sharpens the Job Demands-Resources model and Social Exchange Theory [1,5] rather than merely relocating them. Empirically, it repositions socio-cultural influence as a distal, selection-stage determinant, which explains why organization-level interventions, rather than normative appeals, are where practitioners have leverage. For organizations in Bangladesh, the implication is direct: engagement is built by what managers do and what schedules permit [17,19,22], and career development pays off only when the systems behind it are real. These conclusions are associational and drawn from a cross-sectional, non-probabilistic sample, and warrant longitudinal replication.

### Limitations and future research directions

First, the sample is non-probabilistic and drawn through purposive recruitment via organizational and professional channels; the findings describe associations within this sample and cannot be generalized to the population of employed women in Bangladesh [32,37]. Second, the design is cross-sectional, so causal direction and temporal ordering cannot be established, and reverse causality cannot be excluded: more engaged women may perceive their leaders and culture more favorably rather than the reverse. Third, the socio-cultural scale mixes internalized normative expectation with perceived organizational bias [4,8,14], and this heterogeneity may attenuate its estimated coefficient; disaggregating the construct is a priority for future work. Fourth, although quota targets secured adequate cells for the descriptive sample characterization, the continuous operationalization of our moderators in the PLS-SEM interaction tests means that categorical cell sizes did not limit the statistical power or precision of our estimates, which are reported with full confidence intervals. Fifth, the geographical focus on Bangladesh may restrict the generalizability of the findings to other regions or socio-cultural contexts. Sixth, the reliance on self-reported data introduces the potential for bias, as participants may have provided socially desirable responses or interpreted engagement differently based on individual experiences.

Future research should expand the scope beyond Bangladesh to explore women’s employee engagement in diverse geographical regions and industries. Conducting longitudinal studies would help capture the evolving nature of employee engagement, especially in response to changing organizational policies and socio-cultural influences. Additionally, exploring specific mediating factors, such as age, education level, or professional experience, could offer a more nuanced understanding of how different groups of women experience engagement. Disaggregating socio-cultural influence into internalized norms and perceived bias, and testing the selection-stage interpretation advanced here on a sample that includes women outside formal employment, would provide a direct test of the mechanism proposed in this study. The role of digital transformation and the increasing prevalence of remote work, particularly in a post-pandemic context, presents another promising avenue for future research, as these factors may significantly alter the landscape of women’s engagement in the workplace.

## Supporting information

S1 Data Anonymized item-level survey responses (n = 242) with sector and organizational size categories as “Responses Data” in an Excel file. (XLSX)

## Abbreviations

AVE: average variance extracted
CB-SEM: covariance-based structural equation modeling
CD: career development opportunities
CR: composite reliability
HTMT: heterotrait-monotrait ratio
ICT: information and communication technology
IS: perceived industry gender-equity support
JD-R: Job Demands-Resources
LM: leadership and managerial support
MNC: multinational corporation
OC: organizational culture and policies
OS: perceived size-related HR infrastructure
PLS-SEM: partial least squares structural equation modeling
SC: socio-cultural influences
SME: small and medium-sized enterprise
SRMR: standardized root mean square residual
UWES: Utrecht Work Engagement Scale
VIF: variance inflation factor
WE: women’s employee engagement
WI: work-life integration
